# Outcomes of Elongated Styloid Process Syndrome Treated with Minimally Invasive Cervical Styloidectomy (MICS)—A Single-Center Retrospective Study

**DOI:** 10.3390/jcm13216409

**Published:** 2024-10-25

**Authors:** Jakub Bargiel, Michał Gontarz, Krzysztof Gąsiorowski, Tomasz Marecik, Grażyna Wyszyńska-Pawelec

**Affiliations:** Department of Cranio-Maxillofacial Surgery, Jagiellonian University Medical College, 30-688 Cracow, Poland; michal.gontarz@uj.edu.pl (M.G.); krzysztof.gasiorowski@uj.edu.pl (K.G.); tomasz.marecik@uj.edu.pl (T.M.)

**Keywords:** Eagle syndrome, styloidectomy, stylalgia, MICS, orofacial pain, stylohyoid syndrome

## Abstract

**Background:** Stylohyoid syndrome, also known as classical Eagle syndrome (ES), is a rare condition characterized by elongation of the styloid process (SP) or calcification of the stylohyoid chain, presenting numerous non-specific symptoms. Most papers concerning this rare condition are case reports, case studies, or retrospective studies. This retrospective study delves into the intricacies of Eagle syndrome and evaluates surgical outcomes in patients treated with minimally invasive cervical styloidectomy (MICS). **Methods:** We conducted a retrospective study of patients treated due to ES between September 2021 and August 2023. Ninety-seven patients were qualified for MICS. Evaluation before and after surgical treatment was conducted during follow-up visits and by a questionnaire that included various data, such as symptoms, pain intensity, evaluation of healing period, and overall patient satisfaction. The minimum follow-up period was six months. **Results:** After applying inclusion and exclusion criteria, eighty-four patients were qualified for further assessment. The most common symptoms that subsided completely after treatment were pharyngeal foreign body sensation and pain with dysphagia or odynophagia. The MICS procedure proved to be highly effective, with 94.2% of patients experiencing a significant reduction in pain intensity and 97.1% reporting overall success. All patients were satisfied with the aesthetic outcome of the scar. The length of SP and time onset from the first symptoms to treatment positively correlated with the number, intensity, and time required for symptoms to subside. **Conclusions**: Eagle syndrome should be considered in diagnosing patients with orofacial and cervical pain symptoms, as the time from occurrence of the first symptoms to treatment is crucial. MICS is highly effective and has a low rate of complications in treating ES. Potential scar formation should not be a significant factor when deciding between the intraoral and external approaches.

## 1. Introduction

Elongated styloid process syndrome, commonly recognized as Eagle syndrome (ES), is a medical condition characterized by various neurovascular symptoms, including unilateral cervical and orofacial pain, a sensation of a foreign body in the throat, dysphagia, auditory symptoms (otalgia and tinnitus), and neck discomfort. These symptoms are attributed to the elongation of the styloid process or ossification of the stylohyoid ligament, compressing the adjacent vascular and neuronal structures [1]. Elongation of the styloid process can lead to severe complications, such as carotid artery dissection, which can result in ischemic stroke and other vascular issues [2,3]. Identifying ES is challenging due to its non-specific symptoms, leading to potential oversight by medical practitioners. An elongated styloid process alone is not pathognomonic for Eagle syndrome, as numerous individuals with an incidental discovery of stylohyoid chain anomalies exhibit no symptoms.

The styloid process (SP) is a small bony projection originating from the lower surface of the petrous portion of the temporal bone. The term “styloid process”, originating from the Greek word “στῦλος” (stylos) meaning “stylet”, was first introduced by Galen in the 2nd century A.D. Eagle initially reported that the typical styloid process measures 25 mm. Any length exceeding these values was deemed a contributing factor to Eagle syndrome [4]. Subsequent studies have delved into this subject, using various radiological and morphometric approaches to gain valuable insights. A normal styloid process in an adult typically measures up to 30 mm in length [5]. However, when the styloid process extends over 40 mm, the risk of developing unfavorable symptoms increases significantly [6,7,8].

The styloid process is the attachment point for three muscles and two ligaments, known as Riolan’s bouquet [9]. The stylopharyngeus muscle originates from the medial side of the base of the styloid process and inserts into the posterior border of the thyroid cartilage. It is unique among pharyngeal muscles due to its external origin from the pharyngeal wall. The muscle descends between the external and internal carotid arteries, serving as an important anatomical landmark for identifying vessels in this area. Its primary function involves pulling the nasopharyngeal wall outward while swallowing and breathing. The stylohyoid muscle, emerging from the styloid process’s posterior and lateral aspects, proceeds downward and forward to the hyoid bone’s body near the greater cornu. It assists in elevating the hyoid bone and the tongue’s base during swallowing in coordination with the digastric, mylohyoid, and geniohyoid muscles. The styloglossus muscle originates from the anterior lateral part of the styloid process, close to its apex, and extends downward and forward to insert into the superior and lateral aspects of the tongue. Thus, it functions as an extrinsic tongue muscle, elevating the tongue’s lateral edges and facilitating its retraction when both muscles contract. The stylohyoid ligament, developing from the ceratohyal part of Reichert’s cartilage, spans from the styloid process’s apex to the lesser cornu of the hyoid bone, forming part of the stylohyoid apparatus with the styloid process. The stylomandibular ligament extends from the styloid process’s apex to the mandibular ramus’s posterior border between the masseter and the pterygoid muscles. This ligament supports the action of the styloglossus muscle, contributing to mandibular articulation [9,10].

The stylohyoid apparatus extends inferiorly and anteriorly into the parapharyngeal space, dividing it into retrostyloid and prestyloid compartments. The retrostyloid compartment contains essential structures, including the internal jugular vein (IJV), internal carotid artery (ICA), sympathetic trunk, glossopharyngeal, vagus, accessory, and hypoglossal cranial nerves. The prestyloid compartment contains the external carotid artery, lingual, and auriculotemporal nerves [11].

Involvement of one or more of these structures within the parapharyngeal space or direct contact of the SP with the lateral wall of the pharynx has been associated with various symptoms. The most significant is pain localized in the orofacial and cervical regions.

The treatment of choice for ES is surgical excision of the calcified styloid–hyoid complex, which can be performed via either an intraoral or extraoral approach [12,13]. There is no clear consensus on which technique is superior; however, the intraoral approach has a higher recurrence rate due to incomplete removal of the SP, a greater risk of potential infectious complications, and the lack of technical capability to manage potential vascular complications [14]. The minimally invasive cervical styloidectomy (MICS) is a promising technique that minimizes the risk of surgical complications and improves outcomes [15]. Alternative nonsurgical treatments, including transpharyngeal infiltration of lidocaine or steroids and manual transpharyngeal fracture of the styloid process, have limited outcomes and are typically reserved for patients not eligible for surgical intervention [16].

The main objectives of this study were to assess patients’ response to surgical treatment with MICS and to develop a diagnostic tool to select appropriate candidates for surgical intervention through a comprehensive retrospective cohort study. The secondary goal was to examine the complex symptomatology associated with Eagle syndrome (ES). The insights gained from this research are expected to serve as a foundation for developing more effective therapeutic modalities, ultimately leading to superior patient outcomes.

This study aimed to address the following key questions:

1. What are the predominant symptoms associated with ES?
2. What SP length should alert clinicians to the potential risk of ES?
3. Is MICS effective and safe, making it the optimal choice for treating Eagle syndrome (ES)?

## 2. Materials and Methods

### 2.1. Characteristics of the Study Group

A retrospective analysis of medical records and a questionnaire study of patients operated on due to ES with minimally invasive cervical styloidectomy by the same surgeon at the Department of Cranio-Maxillofacial Surgery, University Hospital in Cracow, Poland, were conducted from September 2021 to August 2023. All patients gave informed consent before surgical treatment and evaluation.

Elongated styloid process syndrome was diagnosed by a comprehensive medical history, detailed head and neck examination, and radiologic findings. The physical examination involved palpating the distal parts of the floor of the mouth, tonsillar fossa, lateral pharyngeal wall, and the region extending from the mastoid apex to the hyoid bone. This aimed to detect a bony structure corresponding to the styloid process or calcified stylohyoid ligament. The elongated SP is not always palpable in the throat due to factors such as insufficient length, trajectory, or anatomical conditions like enlarged palatine tonsils. In cases where palpation of the bony structure is not feasible, patients may still experience significant discomfort in the area of the stylohyoid ligament. Some patients presented with panoramic radiographs and cone beam computed tomography (CBCT). If the initial assessment indicated the likelihood of ES, the patient was referred for further testing. Neck computed tomography angiography (CTA) was the primary diagnostic tool used to confirm the presence of stylohyoid chain anomalies and to assess vascular anatomy variability for surgical planning.

**Figure 1:**
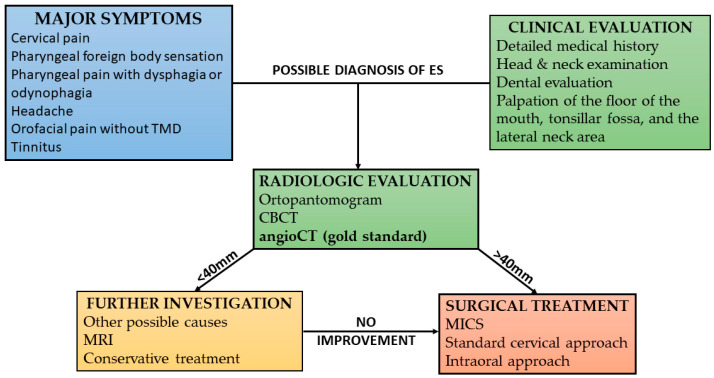
Our diagnostic protocol for patients with Eagle syndrome.

Based on our clinical experience, we used our protocol for diagnosing patients with Eagle’s syndrome (Figure 1).

Patients diagnosed with symptomatic elongation of the styloid process without vascular complications such as carotid dissection or internal jugular vein thrombosis, who underwent assessment of vascular anatomy via CTA and subsequently underwent MICS, with a documented follow-up of at least six months, were included in the study.

**Figure 2:**
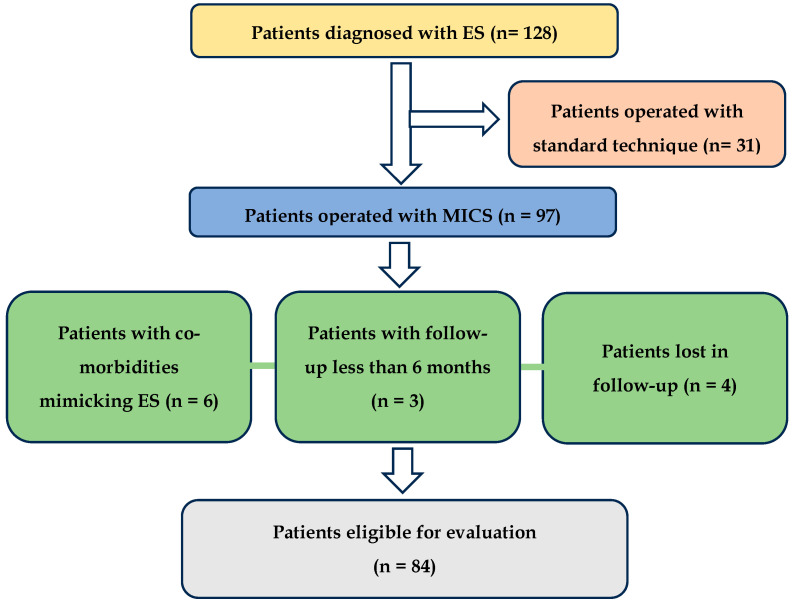
Flowchart of patient selection.

In the study, 128 patients were initially identified, with 97 undergoing MICS surgery. The remaining patients underwent styloid process resection to address vascular issues such as carotid artery dissection or jugular vein stenosis. This was performed via a wider neck approach to improve visibility, protect the neck vessels, and facilitate delicate piezosurgical or diamond burr resection, thereby minimizing the risk of damage to the vein or artery walls. Patients who underwent MICS and presented with head and neck conditions that could mimic the symptoms of elongated styloid process syndrome—such as Kimmerle anomaly, temporomandibular joint disorders (TMDs), and degenerative disk disease, as well as migraines, trigeminal, sphenopalatine, and glossopharyngeal neuralgia unresolved by conservative treatment—were excluded from the study. Individuals who did not return for further outpatient evaluation (lost to follow-up) were also excluded. After applying the inclusion and exclusion criteria, 84 patients were deemed eligible for the study, as illustrated in the flowchart (Figure 2).

Among the selected patients, ages ranged from 16 to 70 years, with a mean age of 39.4 years. The gender distribution was predominantly female, with 64 female patients (76.2%) and 20 male patients (23.8%). The site of the styloid process was nearly evenly distributed, with 41 patients (48.8%) having the styloid process on the right side and 43 patients (51.2%) on the left side. Additionally, 15 patients (17.6%) presented with bilateral stylalgia. The length of the styloid process varied, with the right styloid process ranging from 3.1 to 8 cm (mean 4.9 cm), the left styloid process ranging from 2.4 to 7.4 cm (mean 4.7 cm), and an overall styloid process length ranging from 2.4 to 8 cm (mean 4.8 cm).

The patients’ characteristics are presented in Table 1.

### 2.2. Study Design

This retrospective cohort study was designed to conduct a thorough review of clinical data and perform rigorous statistical analysis to help clarify the complexities of Eagle syndrome. We retrospectively assessed medical records of individuals diagnosed with an elongated styloid process who underwent minimally invasive cervical styloidectomy (MICS) and used questionnaires to follow up on the outcomes. The following data were collected: age, gender, weight, height, body mass index (BMI), preoperative neck CTA with SP measurements, type, site, comorbidities, healing period duration, surgical complications, and subsequent follow-up appointments at the outpatient clinic.

We utilized neck CTA to visualize the elongated styloid process and its relationship to the major vessels. Since a styloidectomy via the neck approach is an operation near the skull base with major vessels in close proximity, the purpose of CTA was to visualize these vessels in high resolution. This allowed for the investigation of anatomical variations (such as kinking or anomalous branches of the internal carotid artery, as well as variations in the external carotid artery branches), the assessment of vascular diseases (including carotid artery dissection, internal jugular vein stenosis, and other vascular abnormalities near the SP), and supported safe surgical planning. While enhanced CT was helpful for visualizing the SP and surrounding soft tissues, it often provided limited visibility of the vessels. The imaging was performed using a GE Healthcare Discovery CT750 HD computed tomography scanner.

**Figure 3:**
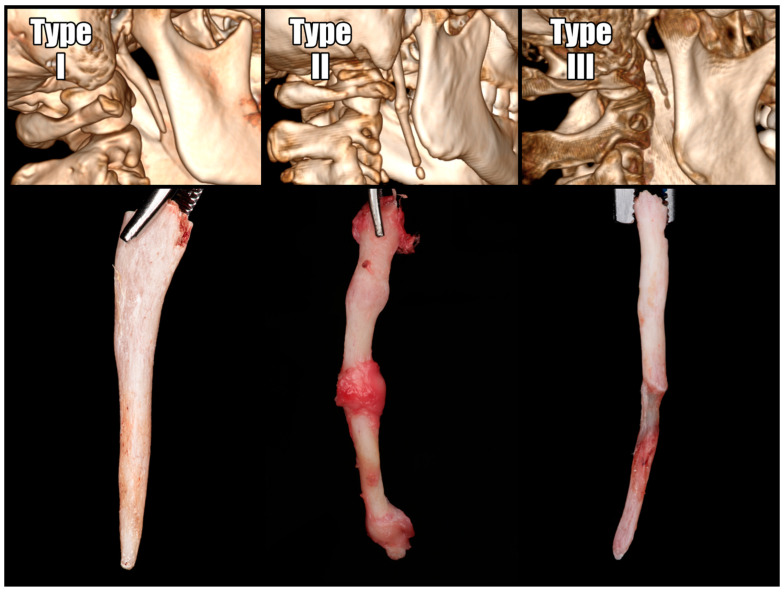
Langlais classification presentation based on our own material. Type I—elongated; type II—pseudoarticulated; type III—segmented.

The lengths of the styloid processes were assessed using the RadiAnt DICOM Viewer software (version 2022.1.1), available at https://www.radiantviewer.com, accessed on 25 September 2024. Measurements were conducted in multi-planar reconstructions (MPRs) and 3D reconstructions to identify the plane that was ideally parallel to the bone’s projection. When the stylohyoid ligament exhibited segmental calcification, the entire length of the stylohyoid chain, extending from the base of the skull, was measured. Operated SPs were classified according to the Langlais classification, which categorizes them based on their structural characteristics [17]. The radiographic appearance of an elongated styloid process and a mineralized stylohyoid ligament can be categorized into three types (Figure 3).

Patient-reported outcomes were evaluated during follow-up visits in the outpatient clinic and by surveys developed by the investigative team using Google Forms. The surveys were designed to assess various aspects of the patients’ experiences.

Pain intensity was measured on a Numerical Rating Scale (NRS) ranging from 0 to 10, with 0 indicating no pain and 10 representing the most severe pain. The scale was assessed before surgery and after a six-month follow-up. The pain levels were categorized as follows: 0—no pain, 1–3—Mild (noticeable but does not interfere with daily activities or is only occasionally distracting), 4–6—Moderate (bothersome, interferes with daily activities), 7–9—Severe (difficult to endure, prevents daily activities, makes normal functioning impossible), and 10—Unbearable (nothing matters except the pain, leading to suicidal thoughts).

Overall treatment satisfaction was assessed using a 5-point scale, where a score of 5 (“very satisfied”) or 4 (“satisfied”) signified success, while scores of 3 (“neutral”), 2 (“disappointed”), and 1 (“very disappointed”) were considered indicative of failure.

To identify the most significant symptoms correlated with ES, the survey included several symptoms in a random pattern in a multiple-choice format. The symptoms reported by at least five patients before surgical treatment were selected for further evaluation. For analysis, non-specific and autonomic symptoms were categorized into three groups:

1. **Visual disturbances**: perception of flashes or flickers of light in the field of vision (photopsia), epiphora.
2. **Cardiovascular symptoms**: palpitations and chest discomfort.
3. **Gastrointestinal symptoms**: sensations of regurgitation and esophageal discomfort.

All data are presented in Table 2.

### 2.3. Treatment

**Figure 4:**
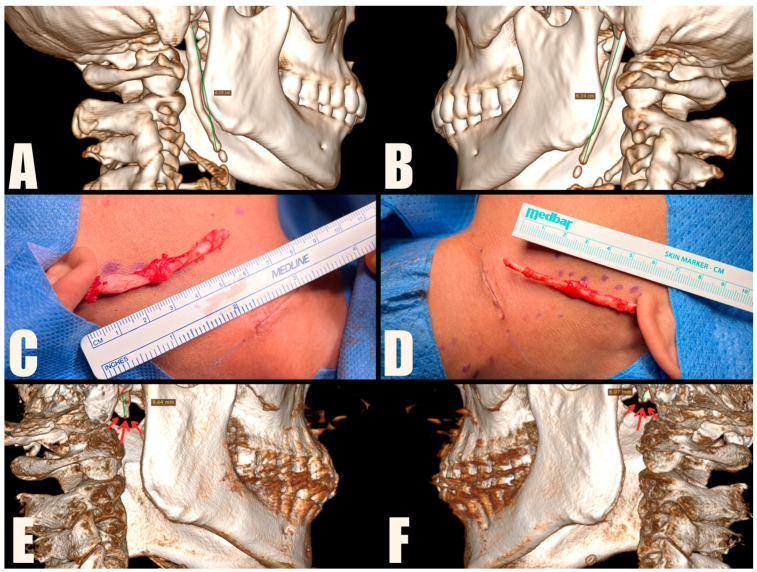
Case presentation of the patient with bilateral ES treated with MICS. (**A**,**B**) Preoperative CT scans, with the SP measuring 6.15 cm and the left SP measuring 6.14 cm. (**C**,**D**) Intraoperative pictures taken after removing the 6 cm long styloid processes. The wounds are sutured with intradermal sutures. (**E**,**F**) Postoperative CBCT scans, indicating residual SP fragments, measuring 9.64 mm on the right and 6.07 mm on the left, highlighted by arrows.

**Figure 5:**
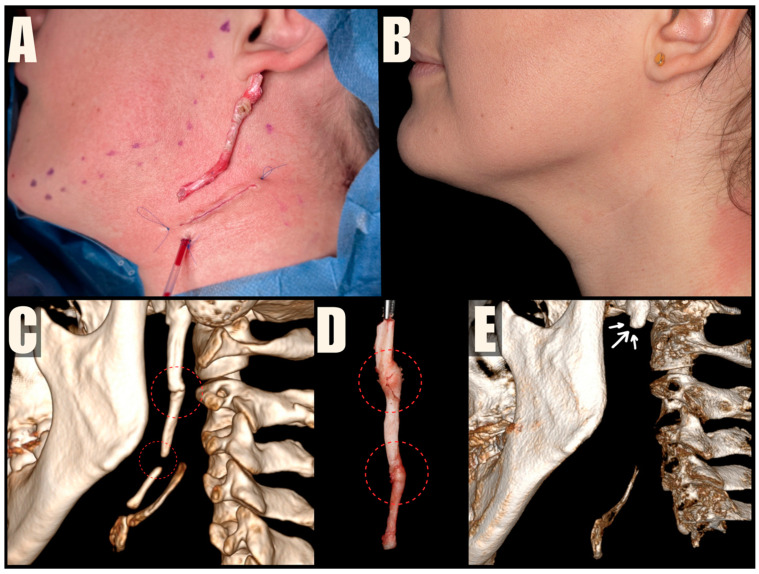
Case presentation of a patient with unilateral ES treated with MICS. (**A**) Intraoperative image of the removed elongated SP, with sutured wound and Redon drainage. (**B**) Scar after 6-month follow-up. (**C**) Preoperative CT scan with elongated SP. (**D**) Removed SP, classified as Langlais type 2; areas of calcification of the stylohyoid ligament marked with a dashed line. (**E**) Postoperative CBCT scan showing residual SP, indicated by arrows.

**Figure 6:**
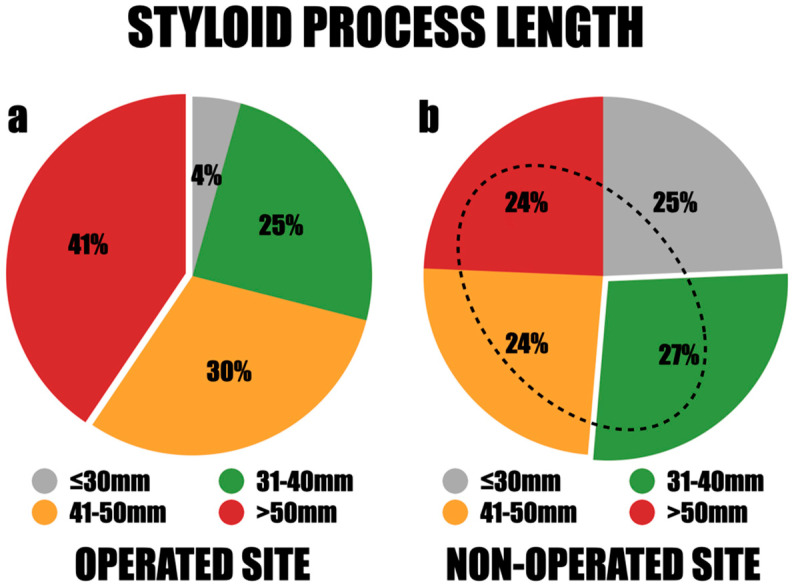
The distribution of styloid process lengths between operated (**a**) and non-operated site (**b**). The dashed line marks the area representing asymptomatic styloid processes measuring over 30 mm.

The MICS procedures were performed under general anesthesia, using magnification loupes and an LED headlamp to enhance visibility. Patients were positioned supine with their heads turned away from the surgeon. The skin was marked to indicate the lower mandibular border, sterno-cleido-mastoid muscle (SCM), and external jugular vein. A small incision was made within a natural skin crease, approximately 3 cm below the mandibular angle, to safeguard the marginal branch of the facial nerve, and 1 cm anterior to the SCM, to protect the greater auricular nerve. The platysma muscle was carefully dissected and divided centrally, preserving muscle bundles for further suturing. A tissue tunnel was created using Pean forceps to reach the base of the skull and expose the sheath covering the styloid process. The styloid process was then carefully cut using Kerrison rongeurs, ensuring a distance of 1 cm from the skull base to protect the facial nerve. The loose styloid process was removed with vascular forceps after detaching muscle attachments. The surgical wound was closed in anatomical layers, including the platysma, subcutaneous tissue, and skin. To improve the aesthetic outcome, the skin was closed with intradermal sutures (Figure 4, Figure 5 and Figure 6). Drains were used sporadically, and antibiotic prophylaxis was not administered.

### 2.4. Statistical Analysis Methods

This study analyzed quantitative variables using descriptive statistics, including mean, standard deviation, median, quartiles, minimum, and maximum values. In contrast, qualitative variables were assessed by calculating absolute and percentage frequencies. The Mann–Whitney U test was used to compare quantitative variables between two groups, and the Kruskal–Wallis test, followed by Dunn’s post hoc test when significant differences were found, was used for comparisons among three or more groups. Spearman’s correlation coefficient assessed correlations between quantitative variables. Qualitative variables in repeated measurements (before and after treatment) were compared using McNemar’s test. A significance level 0.05 was adopted for all analyses, with *p*-values below this threshold indicating significant associations. All statistical analyses were conducted using R software, version 4.4.0.

## 3. Results

Of the 84 eligible patients, 69 agreed to participate in the surveys, resulting in a response rate of 82.1%. Preoperative assessments were based on the total group of 84 operated patients. Postoperative evaluations were based on the group that agreed to further survey evaluation.

### 3.1. Radiologic Data Evaluation

Among the study group, 84 styloid processes were removed, as 15 patients were operated on both sides. Among them, 38 styloid processes (44.9%) were documented on the right side, while 46 (55.1%) were identified on the left. There was a notable disparity in the lengths of these processes, with the longest measuring 80 mm and the shortest measuring 24 mm. The styloid processes were classified into four length categories for further assessment: less than 30 mm, 31–40 mm, 41–50 mm, and greater than 50 mm. Styloid processes exceeding 50 mm were the most frequently observed, accounting for 41%. Those measuring 31–40 mm accounted for 25%, and those measuring 41–50 mm accounted for 30% (Figure 6a). There were no significant differences in length observed between males and females, between both sides, among different age groups, and across various BMI values of the patients. The mean length of the styloid processes was 49.3 mm on the right side and 47.6 mm on the left side. Notably, in all cases where styloid processes were removed bilaterally, the lengths exceeded 40 mm.

The range of styloid process lengths on the asymptomatic side was substantial, with the longest measuring 67 mm and the shortest measuring 22 mm. The length distribution demonstrated that 25% of the styloid processes were less than 30 mm, 27% fell between 31 and 40 mm, and 24% measured 41–50 mm or over 50 mm. The mean length of the styloid process (SP) was 42.8 mm on the right side and 39.2 mm on the left side. Significantly, 76% of the patients had asymptomatic elongation of the styloid processes exceeding 30 mm, and 48% had elongation over 40 mm (Figure 6b).

According to the Langlais classification, the most common group was the solid-structured elongated type I, accounting for 64% of the total. The second most common was the segmented type III, accounting for 22%, while the remaining 14% consisted of the pseudoarticulated type II. The length of the processes on the operated sides was significantly greater in the pseudoarticulated and segmented types (type II and III) compared to the solid type (*p* < 0.05).

There was no statistical relationship between the length and type of the styloid process and other factors such as the patient’s age, pain intensity before surgery, weight, height, and BMI. However, the length of the styloid process determined the resolution of symptoms, indicating that the longer the styloid process, the longer it takes for symptoms to resolve (*p* < 0.05).

### 3.2. Path to Diagnosis

Before patients found the right path toward an accurate diagnosis, they visited various medical specialists, including physicians, dentists, physiotherapists, and osteopaths, for weeks or even years. Although the majority, 38 patients (55.1%), received their initial diagnosis from a consulting physician or a dentist, 30 of them (43.5%) initially identified their potential diagnoses through online resources, such as websites and social media. The final diagnosis was made by oral and maxillofacial surgeons (OMFS) in 40 cases (58.0%). Dentists confirmed Eagle syndrome (ES) in 14 cases (20.3%), and ear, nose, and throat specialists (ENTs) confirmed it in 9 cases (13.0%). Specialists in other medical fields made the remaining 8.7% of diagnoses.

### 3.3. Medical History and Comorbidities

Patients qualified for surgery were in good general health with no significant comorbidities, classified as American Society of Anesthesiologists (ASA) I and II for those older than 65. Regarding BMI, 18 patients (26.1%) were overweight, and two (2.9%) were obese. Only two patients (2.9%) had a history of tonsillectomy. Additionally, a familial occurrence of the disease was observed in three patients (4.3%). Notably, no significant head or neck trauma was reported among the patients. There was no statistical correlation between the influence of factors such as comorbidities, BMI, history of tonsillectomy, or familial occurrence on the disease measured by number of symptoms, pain intensity, and the outcomes of the surgery during the follow-up period.

### 3.4. Time from First Symptoms to Surgical Intervention

The time from symptom onset to diagnosis ranged from 2 to 300 months, with an average of 3.3 years. The duration of the disease demonstrated a statistically significant (*p* < 0.05) and positive correlation (r > 0) with the number of symptoms before treatment. Symptoms such as burning, sensory disturbances, or pain in the back of the tongue; difficulty speaking or hoarseness; dizziness or vertigo; toothache; and visual disturbances were most frequently observed in patients with a disease duration of over four years. In contrast, these symptoms were least common in those whose disease lasted up to 12 months (*p* < 0.05).

### 3.5. Comparison of Symptoms Before and After the Surgery

Patients exhibited a range of symptoms, including the following (given in order): cervical pain, pharyngeal foreign body sensation, pharyngeal pain with dysphagia or odynophagia, headache, orofacial pain, tinnitus, dizziness or vertigo, toothache, burning, sensory disturbances or pain in the posterior part of the tongue, dysarthria or dysphonia, burning sensation in the mouth, syncope, and paroxysmal cough. Among non-specific and autonomic symptoms, cardiovascular issues were most frequent, followed by visual and gastrointestinal disturbances. Almost all symptoms showed a significant improvement after surgery, except for paroxysmal cough, which was not significant due to its infrequent incidence (*p* = 0.134).

Pharyngeal foreign body sensation and pain with dysphagia or odynophagia were the most indicative symptoms of Eagle syndrome (ES), affecting nearly 70% of patients and almost universally subsiding post-treatment. Headaches and cervical pain, in a similar proportion, improved in approximately 80% of cases after treatment. Dizziness, vertigo, and orofacial pain observed in approximately half of the patients also showed a significant reduction after surgery.

In contrast, tinnitus, which was quite common, occurring in 43.5% of patients, did not resolve in more than 50% of cases after surgical treatment.

Less common symptoms, such as burning sensations, sensory disturbances or pain in the posterior tongue, dysarthria or dysphonia, syncope, gastrointestinal issues, and paroxysmal cough, occurred in 20% or fewer patients, making them less typical for ES.

All data are presented in Table 3.

### 3.6. Pain Intensity Based on NRS Before and After Surgical Treatment and Follow-Up

Before the surgery, pain intensity was reported as very severe or the worst possible by 18 patients (26.1%), severe by 48 patients (69.6%), and moderate by 3 patients (4.3%). Furthermore, 38 patients (55.1%) reported constant pain, while 31 patients (44.9%) experienced intermittent pain. After the follow-up period, only one patient (1.4%) reported no improvement and continued to experience moderate pain, defined as bothersome and interfering with daily activities. The surgical treatment proved to be highly effective, with 65 patients (94.2%) experiencing a significant reduction in pain intensity. Among them, 33 patients (47.8) experienced significant relief, reporting mild residual pain, which is noticeable but does not interfere with daily activities or only periodically causes distraction, and 32 patients (47.4%) were completely pain-free. The time for resolving or decreasing pain intensity varied from 0 to 28 weeks, with an average duration of 5.3 weeks. Statistical analysis showed that the most significant reduction in pain occurred within the first two weeks (*p* < 0.004).

### 3.7. Healing Period and Complications

The healing process was uneventful in all cases. There were no inflammatory, permanent sensory complications or issues with scar formation. The intradermal sutures were removed between the 7th and 10th day after the surgery. Temporary ipsilateral facial nerve weakness, caused by compression from a Langenbeck hook during surgical procedure, was noted in two cases (2.9%) but resolved spontaneously within four weeks. Most patients, 40 out of 69 (57.9%), resumed their daily activities or work within the first two weeks. Eleven patients (15.9%) were on leave for two to four weeks. Two patients (2.9%) needed sick leave from one to three months, and only one (1.4%) required leave beyond three months due to severe pain that did not resolve after surgery and follow-up.

### 3.8. Overall Patient Satisfaction

The surgical outcome was measured using three primary parameters: the reduction in pain intensity to a score of 3 or below on the Numeric Rating Scale (NRS), meaning a decrease in symptoms allowing for normal functioning (subjective analysis), and aesthetic satisfaction with scar formation. Sixty-three patients (91.3%) reported being very satisfied, and four (5.7%) indicated satisfaction. Only two individuals (2.9%) remained neutral, stating that the surgical treatment neither met their expectations nor caused harm. Notably, no patients selected options 1 or 2, indicating complete failure. All patients (100%) expressed satisfaction regarding the aesthetic appearance of the scar.

## 4. Discussion

The incidence of Eagle syndrome is controversial. While the literature includes numerous reports on this condition, comprehensive studies analyzing patient demographics, clinical aspects of the disease, and surgical treatment outcomes are still lacking. Incidence rates reported in the literature show significant variation, from as low as 0.09% to as high as 54% across different demographics, highlighting the debate over its actual frequency [18,19,20]. The commonly cited percentage of 0.16% for the exact incidence of Eagle syndrome among all cases of calcification in the stylohyoid chain is based on incorrect assumptions. Eagle’s estimate of a 4% elongation in the population and a 4% symptom occurrence in these patients was based on the author’s assumption [4]. Many studies have retrospectively measured the styloid process in the general population. Although elongated styloid processes were relatively common findings, associated symptoms were not frequently observed [21]. The true incidence of symptomatic Eagle syndrome remains undetermined, highlighting the need for individual patient evaluation.

Three parameters should be evaluated during clinical assessment: medical history, digital palpation of the styloid process, and radiological findings. This can be supplemented by a lidocaine infiltration test, where injecting lidocaine into the tonsillar fossa can provide symptom relief, thereby confirming the diagnosis of Eagle syndrome [22]. During the initial examination, it is essential to note that a typically sized styloid process is not palpable. However, palpation of the styloid process in the tonsillar fossa or the neck area medial to the angle of the mandible may indicate elongation (2). Diagnostic imaging is crucial in identifying styloid chain anomalies, utilizing various techniques such as panoramic radiographs, cone beam computed tomography (CBCT), and computed tomography (CT). A panoramic radiograph, often used in routine dental examinations, can be the first tool to confirm the initial diagnosis [23]. The styloid process should not extend beyond the midpoint of the mandibular body [24]. Magnetic resonance imaging (MRI) can also be considered for a comprehensive assessment of soft tissues, especially in the differential diagnosis of neurologic diseases. Among all modalities, CT is preferred due to its three-dimensional reconstruction capabilities, which allow precise measurements of the styloid process’s length, tip deviation, and proximity to adjacent anatomical structures. The authors recommend CTA for detailed vascular anatomy, which is essential for further surgical planning [15].

Ossification and elongation of the styloid process cartilage begin around the age of 8 and significantly slow by age 30 [25]. The physiological length of the styloid process is typically up to 30 mm [5]. A length exceeding 30 mm is considered elongated, while a length greater than 40 mm is strongly associated with symptoms [8]. In our cohort, 71% of the removed styloid processes were greater than 40 mm, and 25% measured between 30 and 40 mm, supporting this observation. Further analysis of radiologic data and symptoms demonstrated that the length of the SP demonstrated a statistically significant positive correlation with both the number of symptoms and the time required for their resolution. According to the Langlais classification, the length of the symptomatic styloid processes was longer in the pseudoarticulated and segmented types (type II and III) in contrast to the solid type (type I). This suggests a higher likelihood of symptom occurrence in Langlais types II and III. Other measured factors did not correlate with length and type of SP.

Investigation of the non-operated sites revealed that 76% of styloid processes were elongated, measuring over 30 mm, with nearly half of these exceeding 40 mm despite being asymptomatic. Publications indicate that the overall incidence of stylohyoid ligament calcification and styloid process elongation in the population is similar, reaching up to 84.4% and often bilateral [24]. In our study, 15 patients (17.6%) underwent bilateral surgery. There is no clear evidence explaining the cause of styloid process elongation. Given the stylohyoid ligament’s origin from Reichert’s cartilage, it is hypothesized that elongation occurs through ossification or mineralization, which might explain the asymptomatic presence in many patients. Various theories have been suggested to explain the ossification of the stylohyoid ligament. These include reactive metaplasia, congenital dysplasia, trauma-induced loss of ligament elasticity, and degeneration of the tendons [26]. Steinmann proposed an intriguing hypothesis suggesting that a traumatic event can trigger reactive hyperplasia or reactive metaplasia in an initially short styloid process, potentially leading to elongation and ossification of the stylohyoid ligament segments [27]. The real cause of SP elongation remains unknown.

Some authors emphasize the role of genetic predisposition in the inheritance of Eagle syndrome (ES) or suggest trauma as a possible cause [28]. However, in our analysis, there was no history of head and neck trauma, and only one patient reported a familial occurrence. Our study indicates that patients were typically diagnosed in their fourth decade of life, with a BMI in or near the normal range and SP measuring over 4 cm. There was a strong gender predilection, with a female-to-male ratio of 3:1, which is consistent with findings in the literature [29]. The average time from the onset of symptoms to the diagnosis of ES was long. There are no publications concerning this topic. In our material, the time from first symptoms to diagnosis was prolonged, averaging 3.3 years. During that time, the patients visited multiple practitioners, including dentists, general practitioners, ENT specialists, and finally, maxillofacial surgeons who provided the correct diagnosis. It is alarming to note that 43.5% of patients, driven by the severity of their symptoms, found their initial diagnosis of ES on the Internet despite being consulted by medical practitioners. Rapid diagnosis is critical, as a significant positive correlation demonstrated that longer disease duration was associated with a larger number of symptoms.

Irritation of cranial nerves V, VII, IX, or X, the sympathetic trunk, compression of the internal jugular vein, the external and internal cervical arteries, and direct contact with the lateral wall of the throat or the tonsillar fossa are considered to be responsible for the symptoms correlated with ES [30,31,32]. However, there are no clear criteria for which symptoms are typical of ES, leading to confusion in the differential diagnosis. For our investigation to reduce the risk of bias, symptoms were selected based on clinical medical history. Only those reported by at least five patients were included in the evaluation questionnaire. The data identified a clear distinction in the clinical presentation of elongated styloid process syndrome. The most characteristic symptomatic presentation of the disease involved a sensation of a foreign body in the pharynx, throat pain accompanied by dysphagia or odynophagia, and headache and cervical pain, which resolved in the majority of cases. Less common symptoms, such as dizziness, vertigo, and orofacial pain, were observed in approximately half of the patients, showing similar improvement after surgery. However, tinnitus, although frequently reported, persisted in most cases postoperatively. The least common symptoms—burning sensations, sensory disturbances or pain in the posterior tongue, dysarthria or dysphonia, syncope, gastrointestinal disorders, and paroxysmal cough—were significantly more prevalent in patients with a disease duration exceeding four years and least common in those with a disease duration of less than 12 months. These findings suggest that patients who experience a delayed diagnosis not only present with a greater number of symptoms but also exhibit more complex and multifaceted symptomatology. This complexity presents a diagnostic challenge, as non-specific and autonomic symptoms, such as cardiovascular disturbances, visual impairments, and gastrointestinal issues, may mimic the involvement of multiple organ systems. The significant improvement in nearly all symptoms, except tinnitus and paroxysmal, caught following surgical intervention highlights its effectiveness.

Pain is the most common symptom that inflicts the quality of life and causes patients to search for a diagnosis [33]. The direct source of pain in ES remains unclear, with several hypotheses being investigated. The historical hypothesis proposed by Eagle, which linked post-tonsillectomy stretching and fibrosis to the development of Eagle syndrome (ES), has been largely debunked. Contemporary evidence indicates a minimal correlation between tonsillectomy and the onset of ES symptoms. Studies reveal that only a tiny fraction of ES patients have undergone a tonsillectomy shortly before symptom onset, and many have never had the procedure [34]. In our study, only two patients had previously undergone tonsillectomy.

Based on the Visual Analog Scale for pain assessment, 26.1% of the patients in our study reported pain intensity as the worst possible, severe in 69.6%, and only 4.3% as moderate. The nature of the pain was evenly distributed between constant and intermittent types.

Multiple mechanisms have been proposed to explain the pain associated with Eagle syndrome (ES), such as direct irritation of the lateral pharyngeal wall by the elongated styloid process [35], compression of neural structures such as the glossopharyngeal nerve, the lower branch of the trigeminal nerve, and/or the chorda tympani by the elongated styloid process [36], fracture of the ossified stylohyoid ligament or elongated styloid process, leading to granulation tissue formation [37], direct contact between the styloid process and the external wall of the carotid vessels, irritating the sympathetic nerves in the arterial sheath [2], and insertion tendinosis of the stylohyoid ligament that may arise during or after neck activity [38].

Treating Eagle syndrome is divided into conservative and surgical treatment. The treatment method depends on the patient’s symptoms and comorbidities. Surgery is considered the gold standard for treating ES [39,40,41]. In cases where treatment is generally impossible, conservative treatment can be considered. Several authors advocate for initiating conservative management with analgesics, including non-steroidal anti-inflammatory drugs (NSAIDs), and a multimodal pharmacological approach involving anticonvulsants, antidepressants, and local anesthetic injections [42]. The intraoral approach can be executed with endoscopy or robotic surgery, with or without tonsillectomy, depending on the angulation of the tip of the styloid process [43]. The intraoral route has several benefits, including a more straightforward and less time-consuming surgical technique and avoiding a visible cervical scar. However, there are significant drawbacks, such as the necessity of a fluid diet during the healing period, dysphagia, the risk of deep cervical infections due to oral bacterial contamination, poor visualization of the surgical field, and an increased risk of vascular trauma [10]. An inappropriate fracture of the elongated styloid process also poses a risk of bone fragment displacement, making its removal technically challenging [44]. External styloidectomy can be performed using various approaches, most commonly the cervical approach. The primary advantages of the cervical approach include maintaining an aseptic surgical field with minimal risk of surgical site infection and providing adequate exposure to the styloid process and adjacent structures. The main disadvantage is the potential risk of damaging the facial nerve, which can be minimized with a nerve stimulator or maintaining the correct dissection plane. In the MICS technique, the repeatability of following the same dissection plane allows for reliable access to the styloid process without the use of nerve monitoring or the need for any vessel ligation [15]. Small incisions and delicate blunt dissection allow quick recovery with good aesthetic results. In our study, all patients (100%) expressed satisfaction with the aesthetic appearance of their scars.

The MICS is safe and effective, as the healing process was uneventful in the long term, with no significant inflammatory or sensory complications and proper scar formation. Most patients experienced a swift recovery, resuming daily activities or work within two weeks. Temporary facial nerve weakness occurred in two cases but resolved spontaneously within four weeks. The most significant reduction in pain occurred within the first two weeks following surgical intervention. The surgical intervention was highly effective, with 94.2% of patients experiencing a significant reduction in pain intensity and 97.1% reporting satisfaction with the surgical treatment. This overall success rate is notably higher than in previous studies, where success rates were typically around 80% [45,46].

One of the notable strengths of this study is the use of the minimally invasive surgical technique for all patients with strict inclusion and exclusion criteria to reduce the risk of bias. Neck CTA for preoperative planning provided detailed anatomical details, allowing for the assessment of elongated styloid processes and the variability of vessels, thereby facilitating better execution of the surgery. Despite the promising results, the study has several limitations. The study was conducted at a single center by a single surgeon, which may limit the generalizability of the findings. The study’s retrospective nature introduces potential limitations in data accuracy and completeness, which could affect the validity of the conclusions. Additionally, the potential for selection bias due to the retrospective design may impact the representativeness of the patient population and further limit the generalizability of the findings. Although adequate for initial evaluation, the sample size was relatively small for drawing definitive conclusions. While sufficient for observing immediate and short-term outcomes, the follow-up period does not provide information on the treatment’s long-term efficacy. Lastly, the study did not include a control group, which would have strengthened the conclusions regarding the effectiveness of MICS compared to other treatment options.

Future research should address these limitations by including multiple centers, larger patient cohorts, various surgical techniques, longer follow-up periods, and control groups to provide a more comprehensive evaluation of symptomatology and the efficacy and safety of surgical treatment for treating Eagle syndrome.

## 5. Conclusions

An elongated styloid process does not necessarily indicate Eagle syndrome, as more than 70% of patients exhibit various calcifications of the stylohyoid ligament, while syndromic elongation of the stylohyoid chain is rare, affecting less than 0.01% of patients. The key symptoms that should alert health professionals include a sensation of a foreign body in the pharynx and pain associated with dysphagia or odynophagia. These symptoms should prompt further diagnostic imaging for Eagle syndrome, beginning with an orthopantomographic X-ray or CBCT. Other less typical symptoms, particularly those involving the gastrointestinal and cardiovascular systems, should initially prompt investigation of alternative causes.

A styloid process length of over 40 mm is most commonly associated with Eagle syndrome, while lengths between 31 and 40 mm are less likely to be indicative. For further evaluation, particularly when surgery is being considered, a neck CTA is recommended to visualize the neurovascular anatomy and identify potential issues such as carotid dissection or internal jugular vein thrombosis. These findings may necessitate additional diagnostic procedures, other vascular interventions, and potentially a more extensive surgical approach.

Early identification and surgical management in ES is crucial as the severity and number of symptoms increase over time, adversely affecting affected individuals’ quality of life. Despite consultations with various medical practitioners, the average duration for patients experiencing symptoms and seeking an accurate diagnosis was over three years. Most uncommon symptoms, such as cardiovascular, gastrointestinal, and ocular issues, considered non-specific for ES, typically emerged after four years and were less prevalent when the disease duration was less than twelve months.

Minimally invasive cervical styloidectomy has proven to be a highly effective, complication-free surgical technique for removing an elongated styloid process in patients with Eagle Syndrome. Symptom resolution typically occurred within approximately two weeks after surgery, allowing patients to return to their daily activities. The technique demonstrated a 94.2% effectiveness in pain reduction, with an overall patient satisfaction rate of 97.1%. While some authors emphasize scar formation as a potential drawback of the external approach, this concern should not be viewed as a disadvantage of the technique, as scar outcomes largely depend on the surgeon’s skill. With MICS, all patients positively evaluated the aesthetic results of their scar formation.

This article underscores the need for healthcare professionals to promptly recognize and diagnose the disease to prevent symptom escalation and the use of the minimally invasive technique of surgical intervention to improve patient outcomes.

## Figures and Tables

**Table 1:** Patient’s characteristics.

Patients (n = 84)	
Age in years (mean)	16–70 (39.4)
Gender, n (%)	
Male	20 (23.8%)
Female	64 (76.2%)
Site, n (%)	
Right	41 (48.8%)
Left	43 (51.2%)
Bilateral stylalgia, n (%)	15 (17.6%)
Length of right styloid (mean)	3.1–8 cm (4.9 cm ± 14.22 mm)
Length of left styloid (mean)	2.4–7.4 cm (4.7 cm ± 14.66 mm)
Length of styloid in general (mean)	2.4–8 cm (4.8 cm ± 14.41 mm)

**Table 2:** Survey data for Eagle syndrome patient-reported outcomes.

Category	Survey Data	Details
**Diagnosis-Related**	**Family history of ES**	Yes/No
	**History of tonsil removal**	Yes/No
	**Medical specialty of the provider who diagnosed ES**	Multiple choice
**Symptom-Related**	**Time from initial symptoms to diagnosis of ES**	Numeric
	**Symptoms before and after surgery (6-month follow-up)**	Multiple choice
	**Pain intensity**	NRS Scale
**Treatment-Related**	**Time from surgery to a noticeable decrease in symptoms**	Numeric
	**Duration of inability to work or engage in daily activities**	Numeric
	**Satisfaction with the aesthetic appearance of the scar**	Numeric (0–5)
	**Overall treatment satisfaction**	Numeric (0–5)
**Non-Specific and Autonomic Symptoms**	**Visual disturbances**	Yes/No
	**Cardiovascular symptoms**	Yes/No
	**Gastrointestinal symptoms**	Yes/No

**Table 3:** Distribution of symptoms before and after surgical treatment. The *p*-value indicates the statistical significance of the reduction in symptoms following surgical treatment and a minimum of a 6-month follow-up period.

Symptoms	Patients with ES				*p*-Value
	n	%	Symptom Resolved	Symptom Persisted	
Cervical pain	50	72.5%	42 (84.0%)	8 (16.00%)	*p* < 0.001
Pharyngeal foreign body sensation	48	69.6%	47 (97.9%)	1 (2.1%)	*p* < 0.001
Pharyngeal pain with dysphagia or odynophagia	45	65.2%	44 (97.8%)	1 (2.2%)	*p* < 0.001
Headache	45	65.2%	36 (80.0%)	9 (20.0%)	*p* < 0.001
Orofacial pain	35	50.7%	29 (82.9%)	6 (17.1%)	*p* < 0.001
Tinnitus	30	43.5%	13 (43.3%)	17 (56.7%)	*p* = 0.001
Dizziness or vertigo	28	40.6%	22 (78.6%)	6 (21.4%)	*p* < 0.001
Cardiovascular symptoms	28	40.6%	21 (75.0%)	7 (25.0%)	*p* < 0.001
Visual disturbances	25	36.2%	17 (68.0%)	8 (32.0%)	*p* < 0.001
Toothache	23	33.33%	15 (65.22%)	8 (34.78%)	*p* < 0.001
Burning, sensory disturbances, or pain in the posterior part of the tongue	17	24.64%	15 (88.24%)	2 (11.76%)	*p* < 0.001
Gastrointestinal symptoms	14	20.29%	10 (71.43%)	4 (28.57%)	*p* = 0.004
Dysarthria or dysphonia	14	20.29%	13 (92.86%)	1 (7.14%)	*p* = 0.001
Burning sensation in the mouth	13	18.84%	8 (61.54%)	5 (38.46%)	*p* = 0.013
Syncope	12	17.39%	8 (66.67%)	4 (33.33%)	*p* = 0.013
Paroxysmal cough	5	7.25%	4 (80.00%)	1 (20.00%)	*p* = 0.134

## Data Availability

Restrictions apply to the availability of these data. Data were obtained from patients treated at the Department of Cranio-Maxillofacial Surgery, Cracow, Poland, and cannot be shared, in accordance with the General Data Protection Regulation (EU) 2016/679.
